# Cecal perforations due to descending colon obstruction (closed loop): a case report and review of the literature

**DOI:** 10.1186/s13256-022-03674-3

**Published:** 2022-12-05

**Authors:** Mohamed Eltayeb Abdelrahman Naiem, Suliman Hussein Suliman

**Affiliations:** Faculty of Medicine, University of Khartoum, Khartoum, Sudan

**Keywords:** Gastrointestinal tract, Large bowel obstruction, Closed-loop obstruction, Epiploic appendage, Cecal perforation

## Abstract

**Background:**

Cases of large bowel closed-loop phenomenon with cecal perforation are extremely rare, especially when extracolonic epiploic appendage and peritoneal bands are the cause. However, sporadic cases exist in the literature with various presentations, but very few occur in patients in the abdomen without a previous scar.

**Case presentation:**

An 89-year-old Sudanese farmer was admitted to the emergency department with 9-day history of generalized colicky abdominal pain, abdominal distension, anorexia, persistent vomiting, and constipation. Given his clinical presentation and assessment, he was diagnosed with peritonitis due to a perforated viscus in a virgin abdomen. Operative exploration revealed an extraluminal left-sided omento–epiploic band that resulted in closed-loop colonic obstruction with secondary multiple cecal perforations. Standard right hemicolectomy with adhesiolysis was done. Postoperative wound infection and hypoalbuminemia were treated, and the patient was discharged on postoperative day 9 on a regular oral diet.

**Conclusions:**

Although this condition is rare, it can be extremely dangerous, requiring prompt investigation and surgical intervention. It usually occurs secondary to raised intraluminal pressure with subsequent ischemia of the cecal wall. Through this case report, we aim to reflect on this rare experience, shedding light on the benign, extracolonic pathologies that can be life threatening or even fatal.

## Introduction

Colonic/large bowel occlusion (LBO) is a critical surgical condition that requires prompt investigation and treatment. Colonic obstruction caused by genuine mechanical blockage must be essentially distinguished from uncomplicated paralytic ileus or pseudo-obstruction. The age-dependent etiology of this surgical emergency can either originate from a mechanical pause in intestinal content flow or colon dilatation in the absence of a pseudo-obstruction/anatomic lesion. Tumors, diverticulitis, severe inflammatory processes, fecal impactions, and volvulus may be the causes. An epiploic appendage band is an infrequent cause of significant colonic closed-loop obstruction, leading to cecal perforation addressed by our case report. Hence, it presents unusual benign but serious causes that may lead to tremendous comorbidities and even mortality if unrecognized.

## Case presentation

An 89-year-old Sudanese farmer was admitted to the emergency department with a 9-day history of generalized colicky abdominal pain, abdominal distension, anorexia, persistent vomiting, and constipation. The pain started in the left iliac fossa and periumbilical region then progressed to be generalized and colicky in nature. It was moderate to severe, mainly aggravated by eating, while partially relieved after vomiting and analgesia. It was associated with fever, anorexia, nausea, and vomiting. Eventually, the pain became diffuse, generalized, severe, and continuous, with abdominal guarding, distension, and absolute constipation for 1 day before the presentation. The systemic review was clear, apart from bilateral lower limb edema.

The patient had a previous history of left-sided and lower abdominal pain but no lower gastrointestinal symptoms, including bleeding. He had no history of weight loss or jaundice. He had a clear medical and surgical background. He was not allergic to any drugs or under any chronic medications.

On examination, he was fully conscious and oriented to time, place, and person. He was not pale or jaundiced. His presenting pulse rate in the ED was 108 beats per minute, normotensive with 130/70 mmHg blood pressure, and good hydration status. Abdominal examination revealed generalized abdominal distension with full flanks, and diffuse tenderness in all abdominal regions with guarding but no rigidity. There was no palpable organomegaly or masses. There were absent bowel sounds suggestive of severe generalized peritonitis and a bowel perforation. A digital rectal examination (DRE) revealed an empty rectum.

Blood analysis was requested which revealed hemoglobin of 12.6 g/dl, TWBCs of 22,000 × 10^3^, mainly neutrophils, adequate platelets count, and C-reactive protein (CRP) of 189 mg/dl (normal value ≤ 3 mg/dl). The liver function test revealed low serum albumin of 2.3 g/dl (normal value 3.5–5 g/dl) with a preserved albumin globulin ratio. The carcinoembryonic antigen (CEA) tumor marker level was checked, as a perforated colorectal tumor was one of our top differentials, but it was within the normal range. Urinalysis and blood electrolytes were normal.

Erect chest and abdominal X-rays showed dilated bowel loops of the small and large bowel, cecal diameter of ≥ 12 cm, and free peritoneal/subdiaphragmatic gas (air under diaphragm).

Considering the patient’s clinical examination, radiology findings, and patient status, severe peritonitis due to large bowel perforation secondary to an obstructing left-sided tumor was the provisional diagnosis.

The colorectal surgeon’s decision was emergency surgical exploration. The contrast-enhanced computed tomography (CECT) of the abdomen was deferred, as it would delay the surgical exploration.

**Fig. 1:**
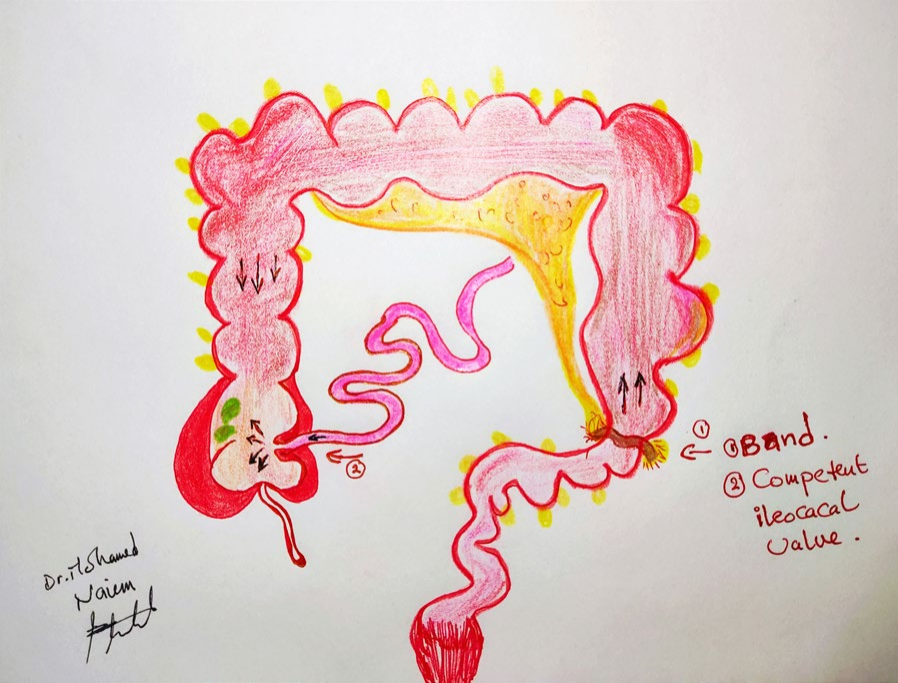
A hand drawing illustrating the closed loop obstruction and mechanism of cecal perforation

Management including intravenous fluoroquinolones and metronidazole antibiotics, fluids, and intravenous human albumin replacement was initiated. An emergency exploratory laparotomy through midline incision revealed a distended large bowel up to the level of obstruction, free intraperitoneal bowel contents, patchy ischemia of the lateral cecal wall with three perforations, and left-sided omento–epiploic extraluminal band causing distal descending colon obstruction (large bowel closed-loop obstruction) (Fig. 1).

**Fig. 2:**
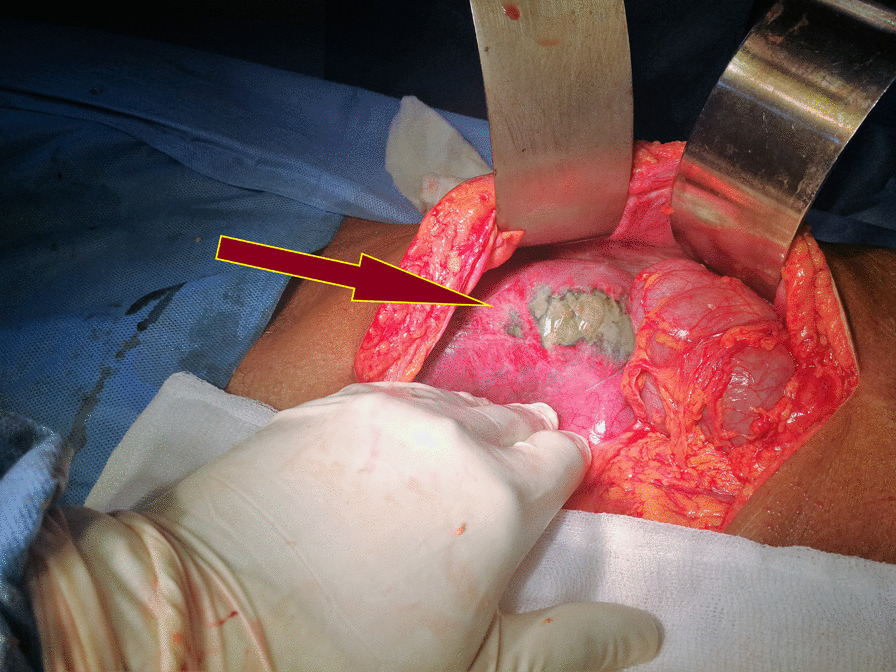
Red arrow: Multiple cecal perforations and dilatation of the cecum

**Fig. 3:**
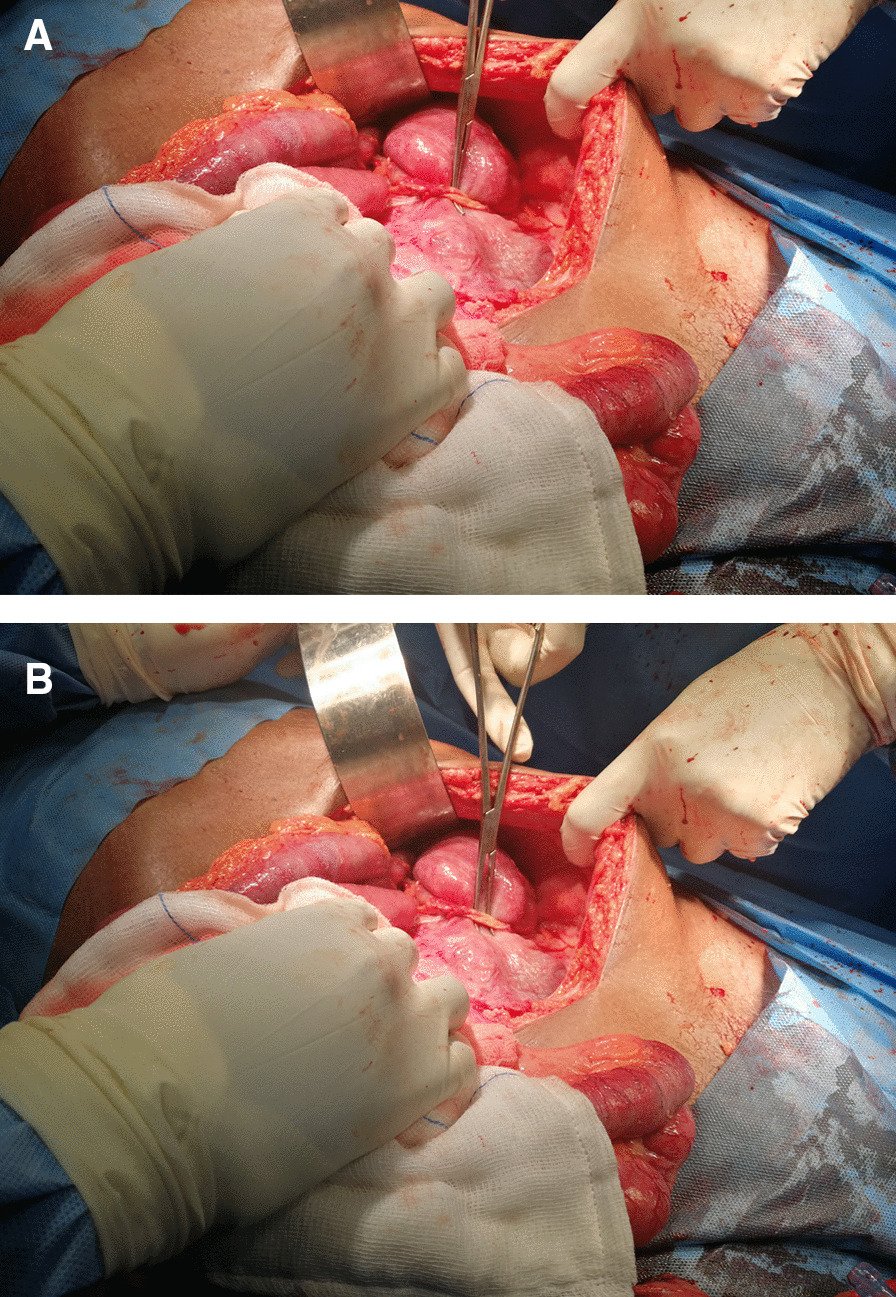
**A**, **B** Both showing the extraluminal band causing descending colon obstruction

Formal laparotomy and careful examination of the abdominal organs, cavity, and bowel were carried out. Furthermore, a simple release of the constricting band with diathermy, standard right hemicolectomy, and ileo-transverse functional end-to-end stapled anastomosis with linear GIA stapler reconstruction (Figs. 2 and 3) was performed. Finally, surgical toilet with warm saline was instilled, pelvic drain was kept *in situ*, and standard mass closure of the abdomen with size one monofilament, nonabsorbable polypropylene suture was accomplished.

Oral intake started on postoperative day 2 and advanced as tolerated by the patient, reaching regular dietary intake by day 7. However, wound infection with purulent wound discharge was discovered during the first dressing on day 3 with intact facial layer. The intraabdominal drain became dry on postoperative day 5.

The patient was discharged on day 9 with wound dressing protocol every other day. Regular dressing was done until the wound was clean by day 14. Surgical stitches were removed during the third postoperative week, and a 3-month follow-up was planned.

## Discussion

Large bowel obstruction (LBO) in elderly patients presenting to the ED represents a complex investigative process considering the broad spectrum of colonic and extracolonic etiologies. Usually, colorectal cancer is the top differential diagnosis for this age group [1, 2]. Epiploic appendages are natural omental pouches filled with fat and distributed over the entire colonic wall to the distal sigmoid colon. Inflammation of these appendages can imitate acute appendicitis or diverticulitis.

We are reporting a rare, exciting case of an extraluminal constricting band on the left side of the abdomen formed by the adherence of the great omentum to the appendices epiploicae of the descending colon. This band resulted in distal large bowel obstruction against a proximal competent ileocecal valve causing massive cecal dilatation, raised intraluminal pressure, and subsequent cecal perforations.

Since this patient had no surgical history of abdominal surgery, with adjunct episode of left-sided lower abdominal pain reported, the possibility of inflammation of epiploic appendages or an occurrence of diverticulitis was the primary suspected cause of the formation of the band [3, 4]. In addition, Rathin *et al*. reported colonic closed-loop obstruction due to adhesions crossing from sigmoid wall appendage epiploicae and attached to the pelvic wall [5].

Although colorectal cancer is the most common cause of large bowel obstruction in this age group, the attending surgeon should consider other causes and work with the patient comprehensively. In the emergency department, the primary imaging modality is the plain erect and supine abdominal X-ray to check for findings suggestive of bowel obstruction and associated complications. Moreover, a CECT of the abdomen is usually requested to know the exact cause and level of obstruction, with the aim of formulating an entire surgical plan preoperatively.

However, the patient’s condition and the expected delay the CECT of the abdomen would cause led the consultant colorectal surgeon to go immediately to emergency surgical exploration. Other differential diagnoses included complicated diverticulitis, volvulus, or internal hernia [6]. Additionally, rare cases exist in the literature in which a transverse colon band and adherence to the right-sided colonic mesentery gave rise to closed-loop obstruction representing an internal herniation [7].

Most of the reported literature on diastatic perforations proximal to an obstructing colonic pathology was related to colorectal cancers. Only three cases were reported for closed-loop colonic obstruction causing cecal perforation in NCBI/PubMed database [8–10].

Operative surgical management is the primary option in cases where frank peritonitis is evident, and conservative strategies can apply in select cases according to patient’s condition and provisional diagnosis. Standard right hemicolectomy or segmental resection and lysis of the band are adequate to maintain the integrity and function of the colon with excellent postoperative outcomes [2, 11].

## Conclusions

Cases of closed-loop colonic obstruction leading to cecal perforations are sporadic, happening because of raised intraluminal pressure with a secondary ischemic cecal wall. The extraluminal band causing this condition is rare and can be seriously dangerous, requiring prompt investigations and surgical intervention.

## Data Availability

The datasets used during the current study are available from the corresponding author on reasonable request. All medical data, supporting materials, and images are available upon request.

## Abbreviations

ED: Emergency department
CRP: C-reactive protein
CEA: Carcinoembryonic antigen
CECT: Contrast-enhanced computed tomography
DRE: Digital rectal examination
WBCS: White blood cells
GIA: Gastrointestinal anastomosis
